# Extracellular vesicles produced by immunomodulatory cells harboring OX40 ligand and 4-1BB ligand enhance antitumor immunity

**DOI:** 10.1038/s41598-020-72122-3

**Published:** 2020-09-16

**Authors:** Isadora Ferraz Semionatto, Soledad Palameta, Jéssica Marcelino Toscaro, Andrea Johanna Manrique-Rincón, Luciana Pereira Ruas, Adriana Franco Paes Leme, Marcio Chaim Bajgelman

**Affiliations:** 1 Brazilian Biosciences National Laboratory, Center for Research in Energy and Materials, Campinas, SP Brazil; 2 Institute of Biology, University of Campinas, Campinas, SP Brazil; 3 Medical School, University of Campinas, Campinas, SP Brazil

**Keywords:** Cancer immunotherapy, Immunosurveillance

## Abstract

Genetically modified tumor cells harboring immunomodulators may be used as therapeutic vaccines to stimulate antitumor immunity. The therapeutic benefit of these tumor vaccines is extensively investigated and mechanisms by which they boost antitumor response may be further explored. Tumor cells are large secretors of extracellular vesicles (EVs). These EVs are able to vehiculate RNA and proteins to target cells, and engineered EVs also vehiculate recombinant proteins. In this study, we explore immunomodulatory properties of EVs derived from antitumor vaccines expressing the TNFSF ligands 4-1BBL and OX40L, modulating immune response mediated by immune cells and eliminating tumors. Our results suggest that the EVs secreted by genetically modified tumor cells harboring TNFSF ligands can induce T cell proliferation, inhibit the transcription factor FoxP3, associated with the maintenance of Treg phenotype, and enhance antitumor activity mediated by immune cells. The immunomodulatory extracellular vesicles have potential to be further engineered for developing new approaches for cancer therapy.

## Introduction

Extracellular vesicles secreted by cells can act as important mediators in the modulation of the immune system^1^. The EVs are a heterogeneous group of membranous structures, of submicron size, secreted by practically all the cells of the organism. They are classified according to their size and subcellular origin. Vesicles produced by budding of the plasma membrane, measuring between 100–1,000 nm, are called microvesicles. In contrast, EVs smaller than 100 nm, generated inside multivesicular bodies (MVE) and secreted after the fusion of the MVEs with the cell surface, are called exosomes^2^. The EVs may carry on their surface numerous proteins inherited from their parent cells, such as tetraspanins CD63, CD81 and CD9, the heat shock proteins Hsp90 and Hsp70 and specific cellular proteins. These proteins may interact specifically with target cells and cause direct stimulation of these cells^3,4^. In this manner, EVs are essential elements for cellular communication, playing a key role in the regulation of multiple biological processes such as pregnancy, blood coagulation, regulation of inflammation and cancer^5–9^.

Tumor cells are large EVs-secreting cells. Although numerous studies evidenced that tumor EVs participate in the progression of cancer, other studies have demonstrated that genetically modified tumor cells can secrete EVs capable of transporting RNA and proteins of interest to target cells, inducing tumor elimination^10–12^. In this work, we investigated the antitumor role of EVs derived from genetically modified tumor cells for expression of 4-1BB and OX40 membrane ligands.

The 4-1BB receptor (also known as CD137 or TNFRSF9) is expressed on activated CD4^+^ and CD8^+^ T lymphocytes, functioning as a potent costimulator of adaptive immune responses. The interaction between 4-1BB and its ligand, the 4-1BBL molecule, induced in activated antigen presenting cells (APCs), stimulates the proliferation, survival and production of pro-inflammatory cytokines by T lymphocytes^13^. Studies with transgenic mice for 4-1BBL protein evidenced a significant increase in the number of memory T cells in these animals^14^. In addition, stimulation of the 4-1BB receptor by agonist antibodies proved to be efficient in reprogramming CD25^+^ CD4^+^ regulatory T cells induced for a cytotoxic effector profile^15^.

The OX40 receptor (also known as CD134 or TNFRSF4) is another costimulatory receptor expressed on the surface of activated T lymphocytes^16^. Co-stimulation of OX40 provides anti-apoptotic signals to T cells^17^. In addition, the treatment of animals with anti-OX40 agonist antibodies is shown to induce the proliferation, differentiation and enhancement of Th1 cytokine secretion profile in CD4+ and CD8+ T lymphocytes^18^. Data in the literature have described the participation of anti-OX40 antibodies in the inhibition of the Foxp3 transcription factor, essential for the maintenance of the regulatory T cells immunosuppressive phenotype^19,20^. OX40 costimulation has been associated to generation of immune memory^21,22^.

We have previously shown that combination of antitumor vaccines harboring TNFSF immunomodulators 4-1BBL, OX40L and the cytokine GM-CSF induce tumor elimination in immunocompetent mice challenged with syngeneic B16 melanoma cells^23^. Moreover, some of these combinations were able to prevent tumor growth in re-challenged mice, suggesting an induction of a long-term protection. Here we explore the immunomodulatory properties of EVs isolated from antitumor vaccines harboring 4-1BB ligand and OX40 ligand.

Tumor cell lines expressing 4-1BBL and OX40L proteins were established by transducing retroviral expression vectors into B16F10 melanoma cells. EVs were isolated and validated for the presence of markers such as CD81, CD63 and CD9. Subsequently, these EVs were tested in vitro in order to assess their immunomodulatory potential. We have demonstrated extracellular vesicles are able to promote changes in the immunological response, mediating the increase of T cell proliferation and inhibition of the immunosuppressive phenotype of regulatory T cells. We also have shown that the immunomodulatory EVs are able to induce the elimination of syngeneic B16 tumor cells in vitro, mediated by splenocytes isolated from C57BL/6. The results of this work have shown the importance of EVs derived from antitumor vaccines, and also suggest the possibility of developing new antitumor strategies based on the engineering of immunomodulatory EVs harboring TNFSF ligands for cancer therapy.

## Materials and methods

### Generation of retroviral vectors

The cDNA of the immunomodulators 4-1BBL and OX40L were amplified by PCR from mice splenocyte and cloned into pCL retroviral vectors, as previously described^24^. The viral preparations were generated and titrated by the Viral Vector Laboratory at LNBio – CNPEM.

### Establishment of cell lines B16F10-4-1BBL, B16F10-OX40L and B16F10-GFP

For the establishment of the B16F10-4-1BBL, B16F10-OX40L and B1610-GFP strains, parental B16F10 cells were transduced with retroviral vectors pCL-4-1BBL, pCL-OX40L and pBabe-eGFP, respectively. The pCL vector encodes an antibiotic resistance gene G418, whereas the pBabe-eGFP vector confers selectivity to puromycin. Resistant clones for G418 were analyzed by flow cytometry using anti-4-1BBL (eBioscience clone RM134L) and anti-OX40L (eBioscience clone TKS-1) clones. Puromycin-resistant clones were also analyzed by flow cytometry.

### Cell culture

The murine B16F10 melanoma cell lines were cultivated in Dulbecco's Modified Eagle's Medium (DMEM) supplemented with 1% penicillin/streptomycin, 1% glutamine and 10% fetal bovine serum (FBS). The EVs from the fetal bovine serum were removed from the medium after 18 h of ultracentrifugation at 110,000×*g* at 4 °C. CD4^+^ T lymphocytes were cultured in Roswell Park Memorial Institute (RPMI) medium, supplemented with 1% penicillin/streptomycin, 10% FBS, glutamine, 1% HEPES, 1% sodium pyruvate, 1% non-essential amino acids and 50 μM β-mercaptoethanol, and maintained at 37 °C in a 5% CO_2_ incubator.

### Isolation of extracellular vesicles

The EVs were isolated according to protocols previously described, with minimal modifications^25^. Briefly, parental B16F10, 4-1BBL and OX40L cells were cultivated in DMEM depleted of FBS-derived EVs, reaching a confluence of 60–80%. Following, the supernatants were harvested every 24 h for 3 consecutive days. The supernatants were centrifuged at 400×*g* for 5 min in order to remove any cellular contents. Subsequently, they were filtered through a 0.22 μm device (Millipore) in order to prevent contamination with cell debris. The ultracentrifugation was performed at 110,000×*g* for 90 min at 4 °C, with the SW32Ti (Swinging-Bucket Rotor-Beckman Coulter) rotor. The EVs pellets at the bottom of the tube were resuspended in sterile saline phosphate buffer (PBS) and stored at − 80 °C. The supernatants were concentrated on the Amicon 100 kDa column (EMD Millipore) and maintained at − 80 °C.

### Nanoparticle tracking analysis (NTA)

The nanoparticle tracking analysis to evaluate the size and concentration of the EVs preparations was performed on the NanoSight NS300 device (NanoSight, Amesbury, United Kingdom). The samples were diluted in 1X PBS to reach a concentration between 10^8^–10^9^ particles/ml and introduced into a chamber equipped with a green laser with wavelength of 532 nm. Video acquisitions were performed on NTA software version 3.1 and the camera settings were appropriately made to provide the correct focus of the EVs. Three videos of 30 s were captured per sample. Both the particle size and concentration were defined as an average of the three videos. These characteristics were determined based on the Brownian motion and the light scattering of the EVs.

### Transmission electron microscopy (TEM) and negative staining of isolated EVs

The EVs preparations were fixed in 2% uranyl acetates and arranged in 400 mesh copper grids coated by an ultra-thin carbon film superimposed on a Lacey carbon film (Ted Pella, Inc., USA). The grids were previously submitted to luminescent discharge of 15 mA/25 s.

The negative staining samples were visualized on the transmission electron microscope (JEOL JEM-1400 Plus) equipped with tungsten filament and operated at 120 kV. The images were analyzed in Gatan Digital Micrograph (GMS3) software.

### Phenotypic characterization of EVs by flow cytometry

EVs were characterized for the presence of CD63, CD81 and CD9 markers. In this protocol 5 × 10^9^ EVs/ml were incubated with 2 × 10^4^ polystyrene beads (Polybeads), adapted from a previously described protocol^26^. The mix of EVs and beads was incubated for 2 h at room temperature and gentle shaking. Then 1 M glycine were added to saturate any free binding sites on the beads surface. Subsequently, the beads were washed twice with 5% FBS PBS and stained with EVs characterizing antibodies. The anti-CD63 (eBioscience clone NVG-2), anti-CD9 (eBioscience clone KMC8), anti-CD81 (eBioscience clone EAT2), and isotype controls IgG2ak- APC (eBioscience), IgG2bk-FITC isotype (BioLegend), and streptavidin-PE. To verify 4-1BBL and OX40L molecules on the surface of EVs, 2 × 10^4^ polystyrene beads (Polybeads) were loaded with capture antibodies anti-4-1BBL (*sc-58949, Santa* Cruz) and anti-OX40L (*MAB1236, R&D*), respectively. Next, beads were saturated with 1 M glycine following incubation with EVs (parental-EV, 4-1BBL-EV, and OX40L-EV). Beads were then labeled with anti-CD9 (eBioscience clone KMC8) and anti-CD81 antibodies (eBioscience clone EAT2) and flow cytometry was performed using Novocyte 2000 flow cytometer.

### Isolation of primary CD4+ T cells and APCs

The CD4+ T cells and APCs were isolated from spleens of C57BL/6 mice. Conventional CD4+ T lymphocytes were immunomagnetically isolated from splenocytes by negative selection (Magnasort Kit, eBioscience). The purity and yield of the separation protocol was verified by flow cytometry and showed greater than 90%. The APCs were purified from murine splenocytes and treated with mitomycin C. Cells were cultured in Roswell Park Memorial Institute (RPMI) medium, supplemented with 1% penicillin/streptomycin, 10% FBS, glutamine, 1% HEPES, 1% sodium pyruvate, 1% non-essential amino acids, 50 μM β-mercaptoethanol, and, the medium was added by IL- 2 (50U/ml). The cells were incubated at 37 °C in a 5% CO_2_.

### In vitro assay to evaluate FoxP3 inhibition

In vitro assay to evaluate the inhibition of the transcription factor FoxP3 was performed by flow cytometry. Briefly, CD4^+^ T cells were incubated in the presence of Inducible regulatory T cell (ITR) cocktail, adding indicated EV preparations. The CD4^+^ T cells were seeded at 5 × 10^4^ cells/well in 96 wells plate and pre-activated with anti-CD3 (Tonbo Biosciences clone 145-2C11) and anti-CD28 (Tonbo Biosciences clone 37.51) 1ug/ml, and after 24 h, it was added ITR induction cocktail (100 U/ml IL2, 1 ng/ml TGF-β and 0.1 ul/ml of retinoic acid). The ITRs were harvested, fixed, permeabilized (FoxP3 Tonbo Biosciences Transcription Factor Dye Kit), labeled with anti-FoxP3-APC monoclonal antibody (eBioscience) and FoxP3 expression was evaluated by flow cytometry. The data were processed in FCS Express 5 Flow Cytometry software.

### In vitro assay to evaluate the induction of antitumor cytotoxicity mediated by immune cells

In this assay, B16F10 tumor cells were incubated with splenocytes and EV preparations. The rational of this experiment was that the EV preparation could induce T cell cytotoxicity resulting in a reduced number of B16F10 cells. Briefly, 1,000 B16F10 cells per well were seeded in 96-well plates, adding same numbers of C57BL/6 splenocytes and EVs preparations at specific concentrations. After 72 h of incubation, the splenocytes were removed and the adherent tumor cells were fixed with 4% paraformaldehyde and stained with DAPI (Sigma). Alive B16F10 tumor cells were analyzed by counting the nuclei stained with DAPI, using as template the wells with tumors cells and media without splenocytes. The quantification was performed by the Operetta HTS Imaging System (PerkinElmer). Each well was evaluated in 75 different fields using 20X magnification. Excitation and emission spectra for DAPI was 460–490 nm and 500–550 nm, respectively. All images were analyzed using the Columbus software (version 2.4.0 PerkinElmer) and representative images were processed with the bio-formats plugin in FIJI.

### Real time quantitative PCR

Each sample was tested in triplicate and all quantifications were normalized to an endogenous control (GAPDH). Total RNA was isolated using Total RNA Purification Kit (CellCo Biotec). We performed cDNA synthesis using the High Capacity Reverse Transcription Kit, (Applied Biosystems). The qPCR amplification was performed and analyzed with ABI Prism 7500 sequence detector (Applied Biosystems). The qPCR reactions were performed using SYBR Green Master Mix (Applied Biosystems) , with 5 µl of template cDNA, 8 pmol of primers, and 10 µl of SYBR Green master mix. The primers used for PCR amplification were: for GAPDH gene: F-AGGTCGGTGTGAACGGATTTG, R-TGTAGACCATGTAGTTGAGGTCA; TGF-beta: F-CTCCCGTGGCTTCTAGTGC, R-GCCTTAGTTTGGACAGGATCTG.

### Statistical analyzes

All data were analyzed using Prism 5.0 (GraphPad software). Statistical significance was assessed by the one-way ANOVA, followed by the Dunnett's test; two-way ANOVA test and T test method, at p-value < 0.05, as indicated in the figures.

## Results

### Tumor-derived genetically modified cells secrete EVs that can be harvested from cell culture media

**Figure 1:**
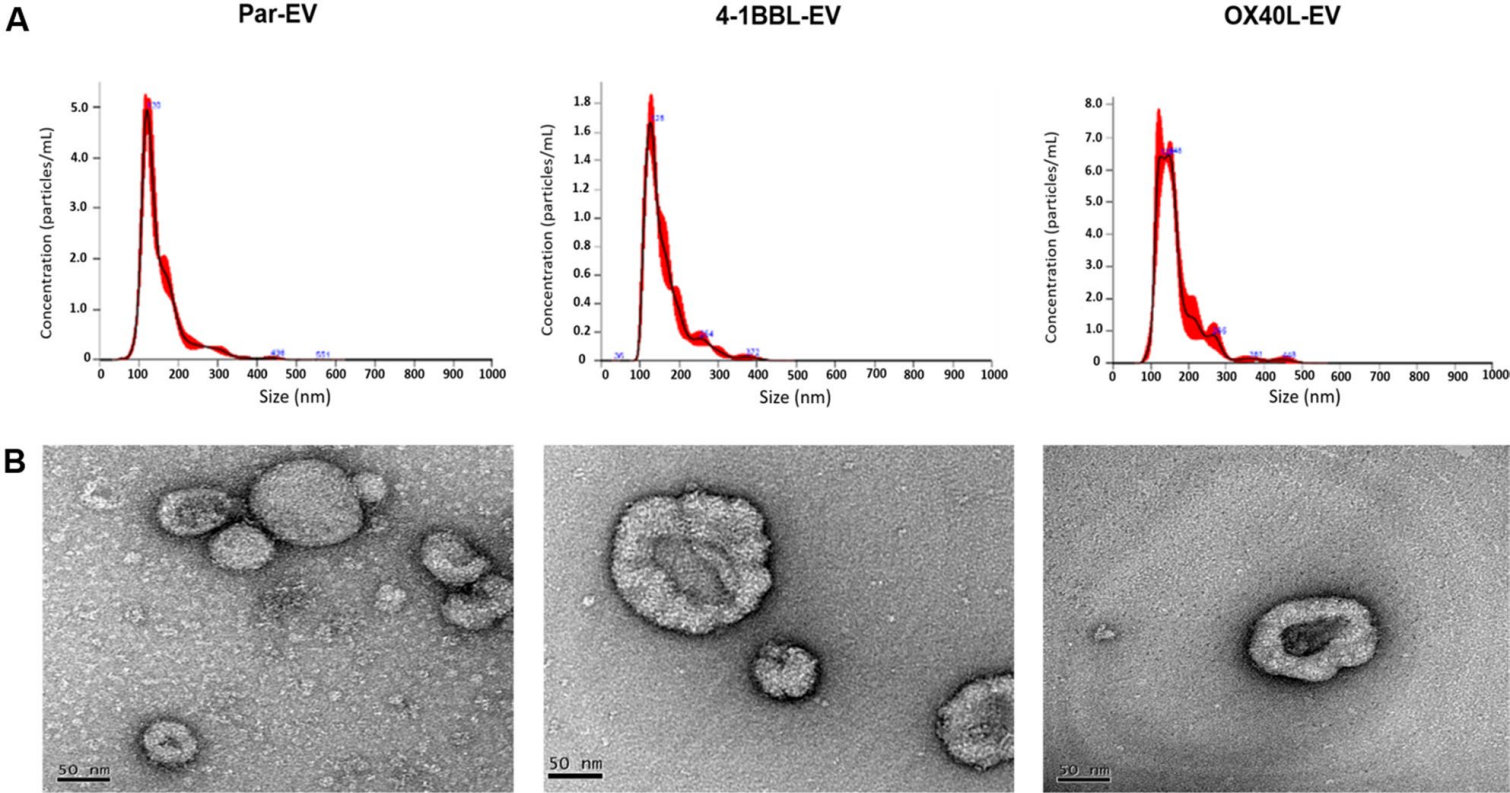
Characterization of EVs using NTA and TEM. (**A**) Representative graphs show NTA results of size and concentration of the EVs. (**B**) Representative TEM images, below each graph, show the isolated EVs from parental-EV, 4-1BBL-EV, and OX40L-EV, using the negative staining method. The NTA images were analyzed by the software NanoSight NTA 3.1 (URL: https://www.malvernpanalytical.com/en/support/product-support/nanosight-range/nanosight-ns300). The TEM images were acquired, analyzed, and processed by Gatan Digital Micrograph (GMS3) software (URL: https://www.gatan.com/products/tem-analysis/gatan-microscopy-suite-software).

We generated antitumor vaccines from parental B16F10 cells that were genetically modified with retroviral vectors encoding the immunomodulators 4-1BBL and OX40L. The expression of these proteins was confirmed by flow cytometry (Figure S1). The EVs were harvested from culture media from parental B16F10 cells, B16F10-4-1BBL and B16F10-OX40L, and characterized using NTA and TEM. In the Fig. 1A, we could observe the mean size of EVs isolated from B16-derived cells was 162 ± 5.31 nm, 155 ± 4.21 nm and 167 ± 7.11 nm, for parental-EVs, 4-1BB-EVs and OX40-EVs, respectively, and concentration was about 1.09 × 10^12^ ± 8.30, 1.96 × 10^12^ ± 9.97, 5.51 × 10^11^ ± 8.87. These preparations were also analyzed by transmission electron microscopy (TEM), confirming the cup-shaped typical morphology of the EVs^27^ (Fig. 1B).

### Extracellular vesicles produced by antitumor vaccines harbor surface immunomodulators

**Figure 2:**
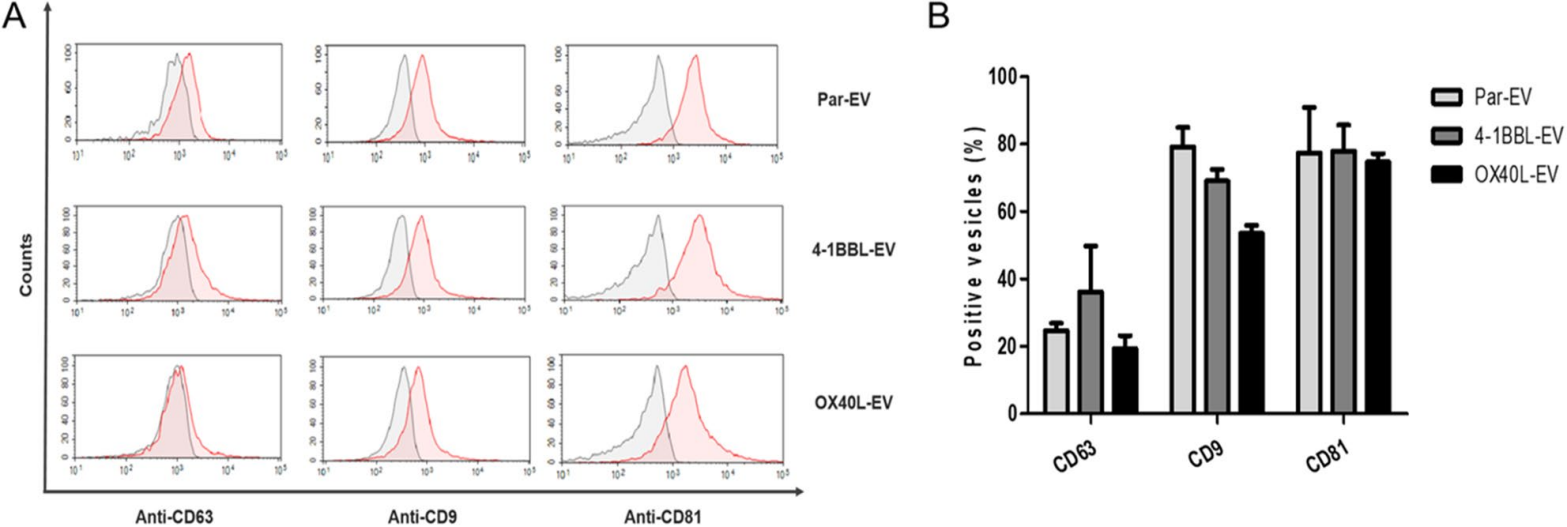
Characterization of EVs isolated from genetically modified B16F10 tumor cells by flow cytometry. The EVs preparations, adsorbed on the beads surface, were labeled with anti-CD63, anti-CD9 and anti-CD81 antibodies. The CD9 and CD81 proteins show greater protein expression in relation to CD63. The difference in the expression of these proteins was evidenced by the shift of the (green) peak to the right, compared to their controls (peak in red), labeled with isotype antibodies, IgG2ĸ. (**A**) In these representative histograms, the y-axis represents the percentage of EV numbers and the x-axis is the mean fluorescence intensity (MFI) on a logarithmic scale. (**B**) Graphic representation of the percentage of positive EVs for each indicated labeling. The bars represent the mean ± SD of three independent experiments. There was no statistical difference of tetraspanins labeling between different groups. Two-way ANOVA test was performed.

**Figure 3:**
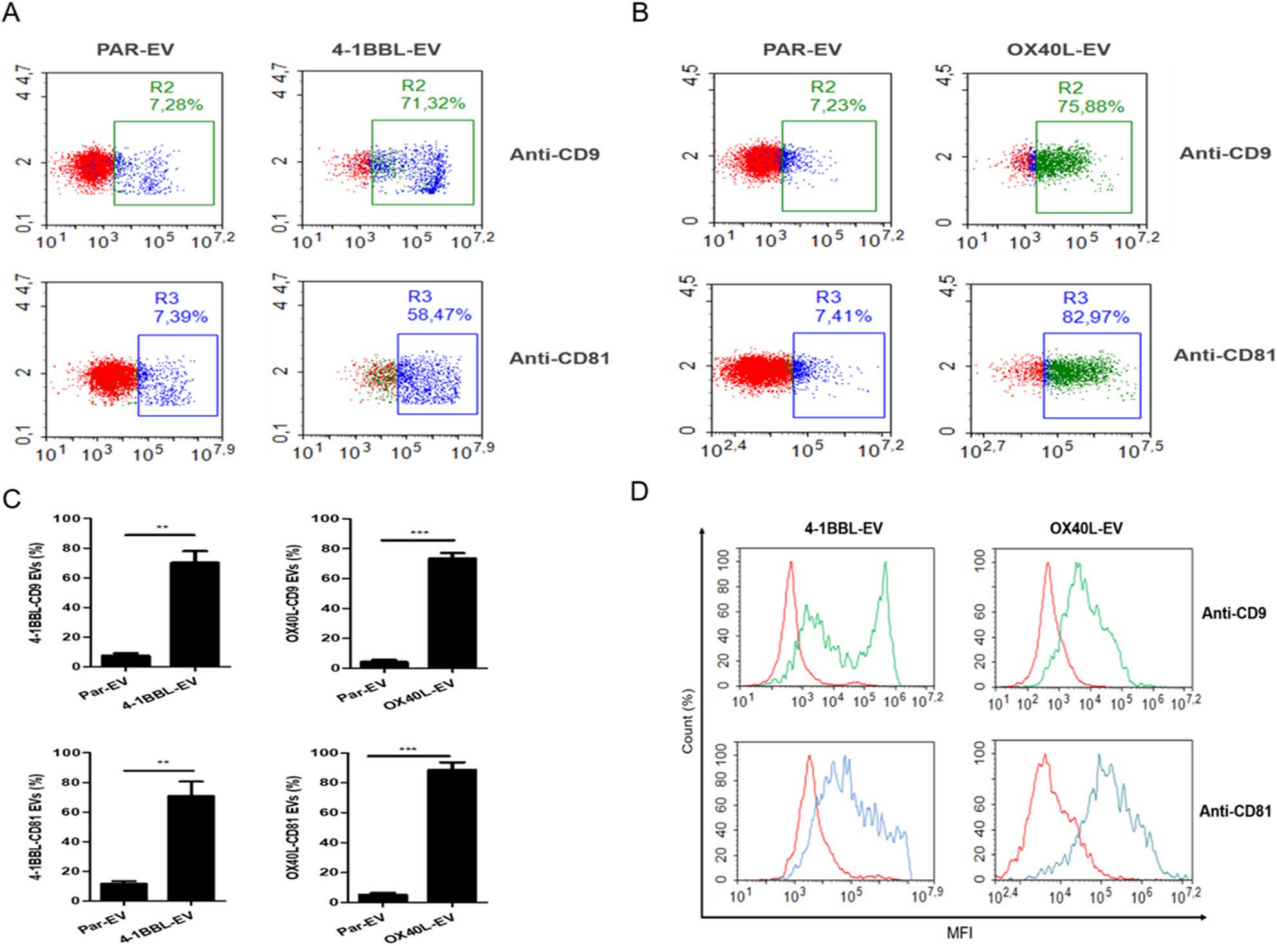
Extracellular vesicles derived from genetically modified tumor cells expressing 4-1BBL or OX40L harbor respective ligands on the surface. We developed a flow cytometry assay in which EVs are captured using polystyrene beads coupled to anti-4-1BBL or anti-OX40L. The EVs bound to beads were subsequently stained for tetraspanins markers CD9 and CD81. (**A**) Dot plot for flow cytometry assay with 41BBL-EVs. (**B**) Dot plot for flow cytometry assay with OX40L-EVs. For all dot plots y-axis is the SSC, and x-axis is FITC intensity for CD9-FITC or APC intensity for CD81-APC. (**C**) Graphic representation of positive EVs for 4-1BBL-CD9, 4-1BBL-CD81, OX40L-CD9 and OX40L-CD81. The bars represent the mean ± SD of three independent experiments. The T test was performed (**p < 0.005; ***p < 0.0001). (**D**) Flow cytometry histogram shows 41BB-EVs and OX40L-EVs. The y-axis represents the percentage of cell numbers and the x-axis is the mean fluorescence intensity (MFI) on a logarithmic scale. Red curves represent parental-EVs used as a control, green curves are EVs preparations positives for CD9 and blue curves are EVs preparations positive for CD81. The figures are representative of three independent experiments.

Since EVs harbor surface markers as CD9, CD63 and CD81^28^, the EV preparations were added to polystyrene beads and stained with fluorescent-labeled antibodies anti-CD63, anti-CD9 and anti-CD81 (Fig. 2). We observed a robust labeling for CD9 and CD81, but low levels of CD63. Next, we developed a flow cytometry-based assay to verify if EVs generated by antitumor vaccines could harbor immunomodulators on their surface. In this assay, beads were loaded with capture antibodies targeting the immunomodulators 4-1BBL or OX40L, following incubation with EVs preparations. The captured EVs were then stained for anti-CD9 and anti-CD81, confirming that the EV harbors both proteins on the EV surface (Fig. 3).

### The B16-derived EVs have the ability to stimulate immune cells

**Figure 4:**
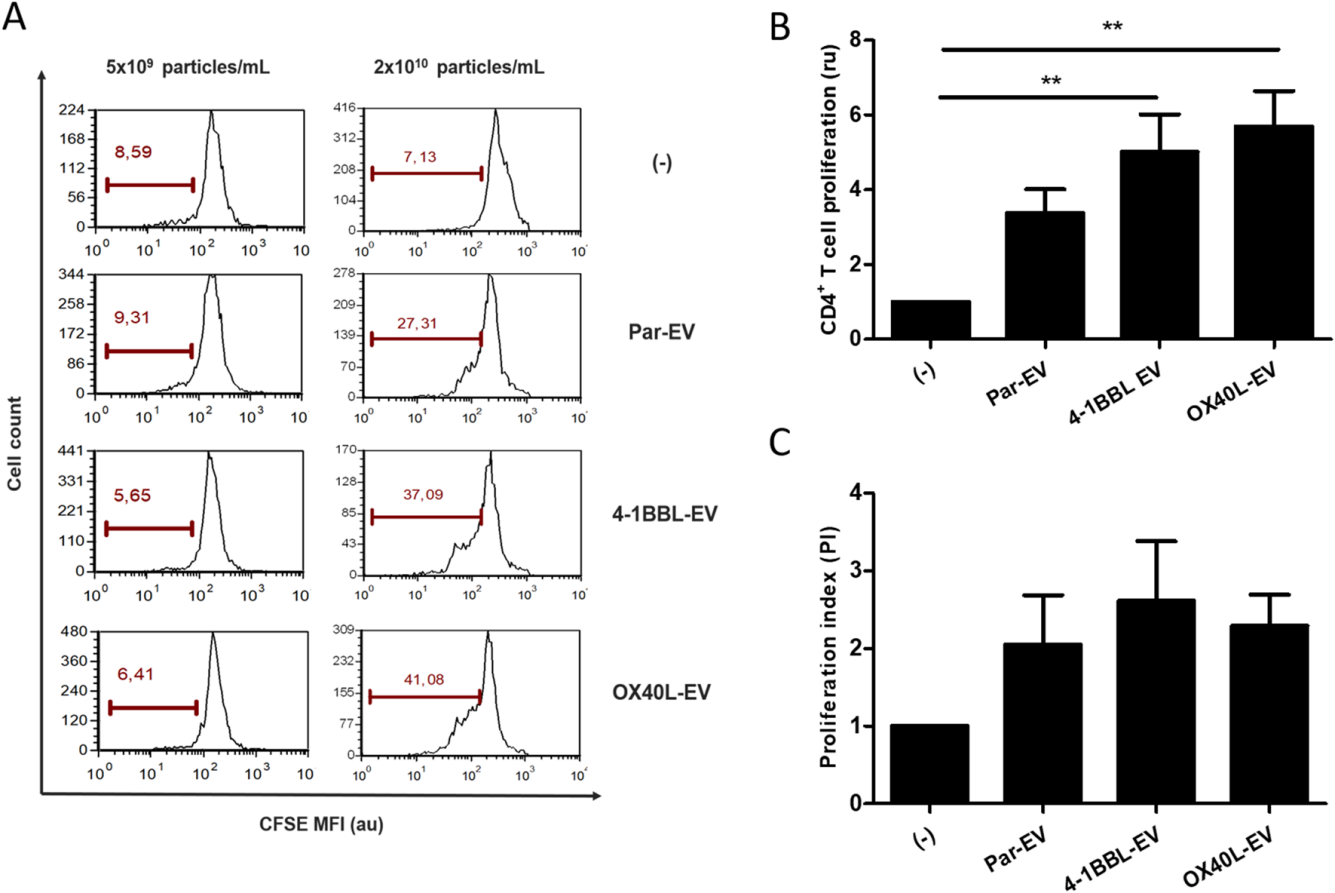
Tumor-derived EVs induce T cell proliferation. CD4+ T cells were labeled with anti-CFSE, incubated with indicated EVs and analyzed by flow cytometry. (**A**) Representative histograms show T cell proliferation. (**B**) Graphic representation to consolidate the proliferation results of three independent experiments. (C) Proliferation index. Data expressed in (**B**) and (**C**) were analyzed by ANOVA with Dunnett post test (**p < 0.05). (–): only CD4+ T cells, Par-EV, parental EVs.

To investigate whether B16-derived EVs may induce biological effects on immune cells, we initially performed a proliferation assay, incubating EVs with primary CD4^+^ T cells isolated from C57BL/6 mice. The CD4^+^ T cells were CFSE-labeled and incubated with indicated EVs preparations. As seen in the Fig. 4, higher EVs concentrations (2 × 10^10^ particles/ml) were able to enhance T CD4^+^ proliferation for all tested conditions.

### The B16F10-OX40L derived EVs have the ability to reduce expression of the FOXP3 transcription factor in vitro

Once we confirmed that EVs could stimulate lymphocytes, we performed an assay to verify if a specific ligand on EV surface could trigger a more specific immunomodulatory signaling on T cells. It has been described that agonistic signaling transduced by an antibody bound to OX40 receptor, which is constitutively expressed on the cell surface of regulatory T cells, induces inhibition of the transcription factor FoxP3 and prevents generation of inducible regulatory T cells^29^.

**Figure 5:**
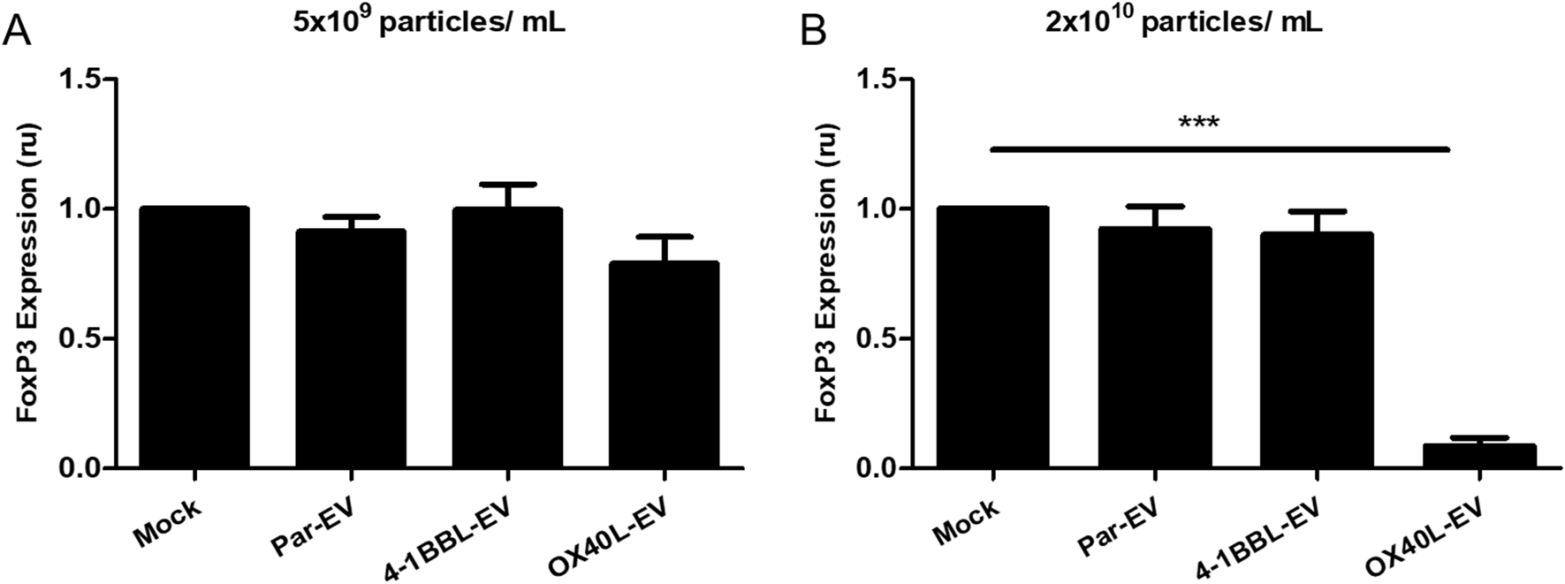
TNFSF-EVs inhibit FoxP3 expression in regulatory T cells. CD4+ T cells were treated with ITR induction cocktail and incubated with the indicated EV preparation. Cells were harvested, stained against FoxP3 and analyzed by flow cytometry. (**A**) 5 × 10^9^ particles/mL and (**B**) 2 × 10^10^ particles/mL. Three independent experiments were performed. One-way ANOVA statistical test followed by Dunnett’s test, *p < 0.05; mean and standard error.

In this manner, to explore immunomodulatory properties for EVs, we performed an in vitro assay incubating immunomodulatory EVs with CD4^+^ T cells that were cultivated in presence of a Treg conversion cocktail, and we could observe that a high concentration of OX40L-EVs induced FoxP3 inhibition (Fig. 5). The FoxP3 inhibition was also associated to a reduction on TFG-β expression (Figure S3).

### The antitumor response is potentiated in the presence of immunomodulatory extracellular vesicles harboring 4-1BBL and OX40L

**Figure 6:**
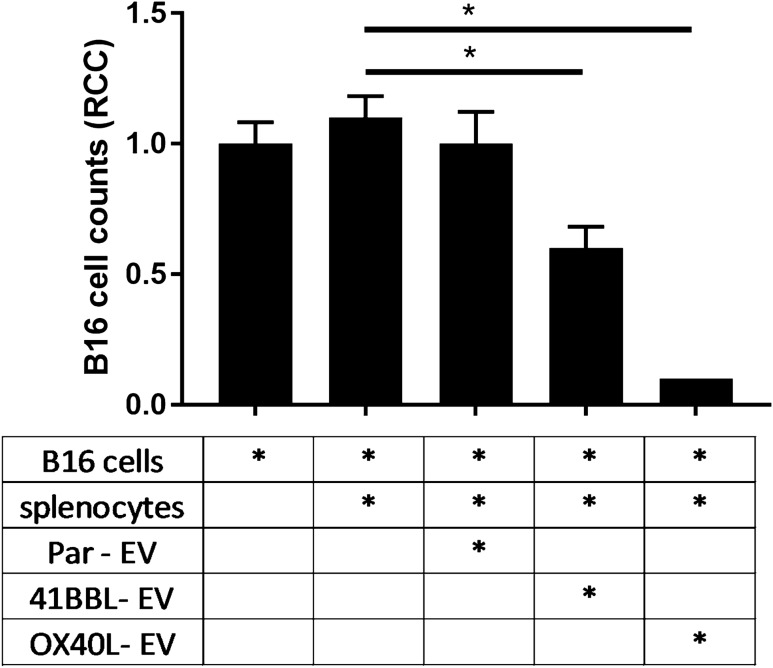
Immunomodulatory EVs induce elimination of tumor cells. In this assay B16- tumor cells were incubated with splenocytes and the indicated immunomodulatory EVs for 72 h, using 2 × 10^10^ EVs/well. Tumor cells were imaged by a high content imaging system (Operetta, Perkin Elmer) and the analyses were performed using the Columbus software 2.4.0 (Perkin Elmer, URL: https://www.perkinelmer.com/Product/image-data-storage-and-analysis-system-columbus). (**A**) B16F10, (**B**) B16F10 that were incubated with splenocytes, (**C**) B16F10 that were incubated with splenocytes and parental-EV, (**D**) B16F10 that were incubated with splenocytes and 41BBL-EVs, (**E**) B16F10 that were incubated with splenocytes and OX40L-EVs. One-way ANOVA statistical test followed by Dunnett’s test, *p < 0.05; mean and standard error. All comparisons were performed against the control of B16F10-cells.

The extracellular vesicles derived from genetically modified B16-4-1BBL and B16-OX40L cells harbor the TNFSF ligands on the surface, and the OX40L-EV was shown to inhibit FoxP3 transcription factor, which may control the immunosuppressive phenotype of regulatory T cells, potentiating the antitumor response. The TNFSF ligands are well known by the agonistic effect on T cells, enhancing its antitumor activity. To investigate the stimulation of antitumor response mediated by immunomodulatory EVs, we performed a High Content Imaging-based assay. In this assay, B16 tumor cells are incubated with freshly isolated C57BL/6-derived splenocyte in the presence of EVs preparations. As seen in Fig. 6 and Figure S2, we observed a significative cytotoxic and antitumor activity of splenocytes on tumor cells using TNFSF ligand-EVs. These results suggested that the 4-1BBL-EV and OX40L-EV are able to induce an antitumor response that was not observed for the parental B16-derived EVs.

## Discussion

In this work we demonstrate that extracellular vesicles derived from antitumor vaccines encoding the expression of 4-1BB ligand and OX40 ligand immunomodulators exhibit immunomodulatory activity. Using flow cytometry assays, we demonstrated the existence of TNFSF ligands on vesicle's surface. We developed a bead-based capture assay in which we immobilized 4-1BBL or OX40L specific antibodies. Since the vesicle harboring these ligands binds to the capture antibody, it can be labeled with a second tetraspanins-targeted antibody, which is normally expressed on the surface of extracellular vesicles, such as CD9 or CD81^4,30^. Therefore, we demonstrate that TNFSF ligands are assembled in the extracellular vesicle and are not soluble proteins in the culture medium. 4-1BB and OX40 signaling induce biological effects associated with increased lymphocyte activity^13^. As previously described, extracellular vesicles may have MHC II molecules on their surface and thus stimulate lymphocyte proliferation in vitro^30^. We observed that vesicles derived from the 4-1BBL and OX40L tumor derived cells have shown a higher costimulatory activity compared to parental vesicles. The costimulation of T cells targeting TNFSF receptors has been widely investigated for cancer therapy. Patients treated with a melanoma-derived antitumor vaccine for 41BB-L expression exhibited an increase in the levels of antitumor CD8 T cells, demonstrating that the 4-1BB costimulation enhances antigen-specific CD8 T cells^31^. Previous data from our group also demonstrated that tumor-derived vaccines expressing TNFSF ligands OX40L and 4-1BBL can generate protective responses in re-challenged animals. Besides enhancing T cell infiltration on tumor sites, the combination of 41BBL and OX40L tumor-derived vaccines have also induced a reduction in Treg infiltrate on tumor sites^23^.

The regulatory T cell has the unique property of inhibiting effector T cell proliferation and may antagonize the antitumor immune response^32,33^. Clinical data show there is a correlation between increased regulatory T cells and a worse prognosis in cancer treatment^34–36^. Thus, inhibition of regulatory T cells may enhance immunosurveillance, boosting detection and elimination of tumors^37–39^. Costimulation via OX40 receptor induces FoxP3 inhibition^29^, which is considered a phenotypic determinant of regulatory T cells^40,41^. We have found immunomodulatory vesicles harboring OX40L ligand induce a significant reduction in FoxP3 expression.

In addition to inhibition of regulatory T cells, the OX40L ligand may also trigger other signaling contributing to antitumor response, such as strengthening lymphocyte activity and longevity^13,42^. Thus, we used an in vitro assay previously developed by our group in which a syngeneic tumor cell line is incubated with splenocytes in the presence of immunomodulatory agents^23^. In the case of antitumor activity, we observed a reduction in the number of cells, by a high-content imaging assay. In this experiment, we found that 41BBL and OX40L immunomodulatory vesicles induced antitumor response, and a more pronounced effect was observed with the OX40L vesicle, which showed a tumor inhibition of about 90%.

In conclusion, we found that vesicles derived from anti-tumor vaccines can harbor immunomodulatory molecules on their surface, which allow T-cell costimulation, regulatory T-cell inhibition and potentiation of anti-tumor immune response. These vesicles can be further engineered to carry other immunomodulatory ligands or even other specific ligands to drive vesicles tropism to tumor sites, thereby generating new tools for cancer treatment.

## Supplementary information

Supplementary information.

## Data Availability

The datasets generated during and/or analyzed during the current study are available from the corresponding author on reasonable request.
