# Supplementary material for: Extracellular vesicles produced by immunomodulatory cells harboring OX40 ligand and 4-1BB ligand enhance antitumor immunity
Source: Sci Rep. 2020 Sep 16;10:15160. doi: 10.1038/s41598-020-72122-3 (PMC7495001; doi:10.1038/s41598-020-72122-3)
Supplement: Supplementary file 1 — Supplementary information. [file 41598_2020_72122_MOESM1_ESM.docx]

**Extracellular vesicles produced by immunomodulatory cells harboring OX40 ligand and 4-1BB ligand enhance antitumor immunity**

Isadora Ferraz Semionatto^1,2^, Soledad Palameta^1,2^, Jéssica Marcelino Toscaro^1,3^, Andrea Johanna Manrique-Rincón^1^, Luciana Pereira Ruas^1^, Adriana Franco Paes Leme^1,2^, Marcio Chaim Bajgelman^1,2,3*^

^1^ Brazilian Biosciences National Laboratory, Center for Research in Energy and Materials, Campinas-SP, Brazil

^2^ Institute of Biology, University of Campinas, Campinas-SP, Brazil

^3^ Medical School, University of Campinas, Campinas-SP, Brazil

* Correspondence: Marcio Bajgelman, email: [marcio.bajgelman@lnbio.cnpem.br](mailto:marcio.bajgelman@lnbio.cnpem.br)

Figure S1


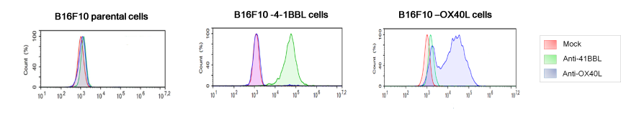


**Figure S1** – Tumor-derived vaccines express TNFSF immunomodulators. B16F10 cells were transduced with retrovirus preparations encoding 4-1BBL or OX40L and selected with G418. Cell pools were harvested, stained with monoclonal antibodies anti-41BBL, anti-OX40L and analyzed by flow cytometry.

Figure S2


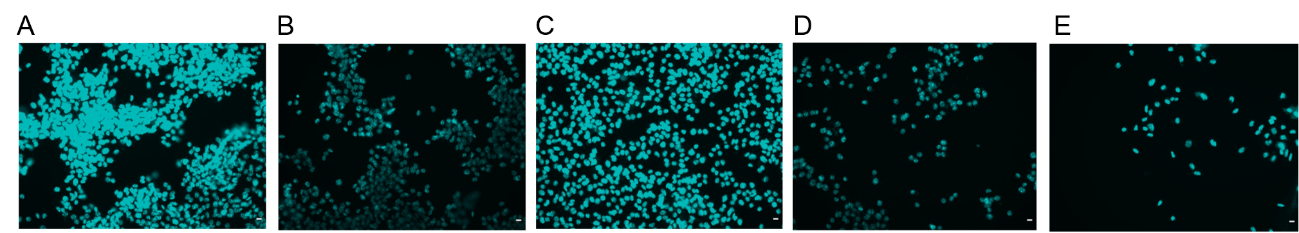


**Figure S2 -** Immunomodulatory EVs induce elimination of tumor cells. Representative pictures of tumor cells that are counted by a high content imaging system (Operetta, Perkin Elmer). (A) B16F10, (B) B16F10 that were incubated with splenocytes, (C) B16F10 that were incubated with splenocytes and parental-EV, (D) B16F10 that were incubated with splenocytes and OX40L-EVs, (E) B16F10 that were incubated with splenocytes and 41BBL-EVs.

Figure S3


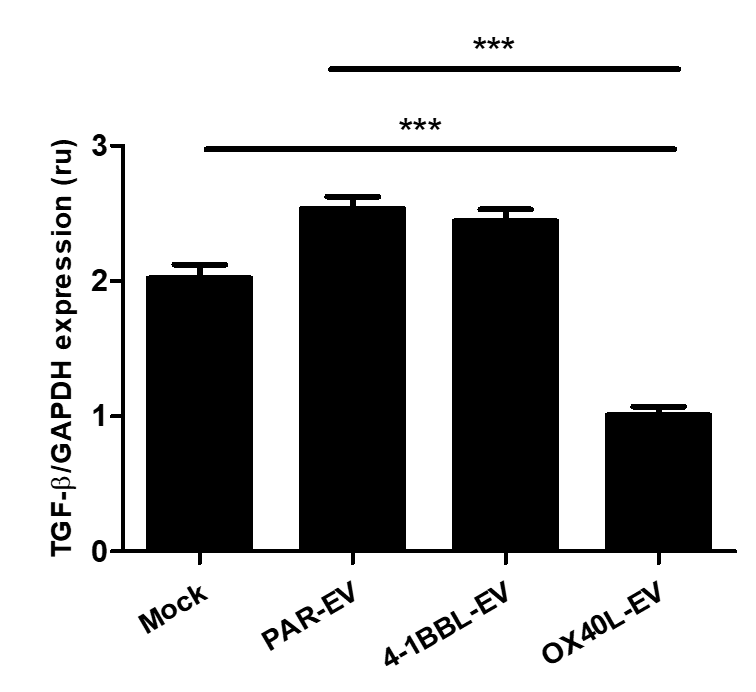


**Figure S3** – OX40L-EV inhibit TGF-beta expression on Treg. Inducible Treg were incubated to the indicated EV, using 2x10^10^ EV per well on 96 wells plates. A qPCR experiment was performed in triplicate using cDNA relative to 50ng of RNA. GAPDH was used to normalize TGF-β expression. One-way ANOVA statistical test followed by Dunnett’s test, * p<0.05; mean and standard error. All comparisons were performed against the control mock and the Par-EV condition.
